# Cocaine-Induced Asthma and the "Crack Lung": A Case Report

**DOI:** 10.7759/cureus.53904

**Published:** 2024-02-09

**Authors:** Victor A López-Félix, Luis A González-Torres, Alan Gamboa-Meza, Gabriela Alanís-Estrada, Juan Francisco Moreno-Hoyos-Abril

**Affiliations:** 1 Internal Medicine, Hospital José Eleuterio Gonzalez, Universidad Autónoma de Nuevo León, Monterrey, MEX; 2 Pulmonary and Critical Care, Hospital José Eleuterio Gonzalez, Universidad Autónoma de Nuevo León, Monterrey, MEX

**Keywords:** persistent bronchospasm, crack lung syndrome, crack lung, acute asthma exacerbation, cocaine ingestion

## Abstract

Cocaine, the second most used illicit drug, is associated with cardiovascular, pulmonary, and other complications. Lung involvement associated with cocaine use, also known as "crack lung syndrome" (CLS), can elicit new-onset and exacerbate chronic pulmonary conditions. A 28-year-old female with a history of chronic controlled asthma arrived at the Emergency Department (ED), referring to cocaine inhalation, followed by symptoms compatible with an asthmatic crisis, requiring immediate steroid and bronchodilator therapy. Radiological studies and bronchoscopy confirmed CLS diagnosis. Despite treatment with oxygen, bronchodilators, and steroids, the asthmatic crises persisted. However, after 48 hours, we observed a complete regression of the lung infiltrates.

This case highlights the importance of clinical suspicion, bronchoscopy findings, and the potential co-occurrence of CLS with asthma exacerbations. While computed tomography (CT) scans can be helpful, they should not be the only tool to diagnose CLS. The successful management of CLS involves the use of bronchodilators, steroids, and oxygen therapy and abstaining from cocaine use. Researchers should conduct further studies to diagnose and treat CLS in conjunction with acute asthma symptoms to assist this patient population better.

## Introduction

Cocaine stands as the third most frequently used illicit drug globally, trailing behind cannabis and opioids. According to the 2021 report from the United Nations Office on Drugs and Crime, the global prevalence of cocaine in the population aged 15-64 was 0.42%. In the United States, the reported prevalence of cocaine use during the 2018-2019 period was 2.14% [1]. Cocaine toxicity is renowned for its association with various complications, including cardiovascular and respiratory issues [2].

The term "crack lung syndrome" (CLS) was coined by Kissner et al. [3] in 1987, documenting a case involving a female patient who exhibited pulmonary infiltrates, airway obstruction, and fever on three separate occasions; these symptoms were promptly resolved following immediate drug interruption. The use of cocaine can cause damage to the lungs due to its effects on the sympathetic nervous system. Cocaine blocks the reuptake of norepinephrine and dopamine, leading to an increase in pulmonary arterial pressure and pulmonary vascular tone, resulting in lung injury, such as pulmonary hypertension, the destruction of the alveolar wall, mucous membrane necrosis, and impaired macrophage function. These mechanisms contribute to the development of cocaine-induced lung injury (CLS). Clinical presentations related to CLS include alveolar hemorrhage, recurrent pulmonary infiltrates with eosinophilia, pneumothorax, pneumomediastinum, pulmonary artery hypertrophy, and thermal injury to the airway. While chest X-rays provide nonspecific findings, computed tomography (CT) chest scans may reveal ground glass opacities, halo signs, lung alveolar infiltrates, and other patterns [3-5].

People who use cocaine are at a higher risk of experiencing asthma exacerbations or similar symptoms. In our case report, we describe a severe bronchospasm in a patient with asthma who had inhaled cocaine. Despite initial treatment failure, the symptoms were ultimately resolved after 48 hours. We aim to highlight the connection between bronchoconstriction and CLS, as demonstrated in our case, and to examine the existing literature on this topic.

## Case presentation

**Figure 1:**
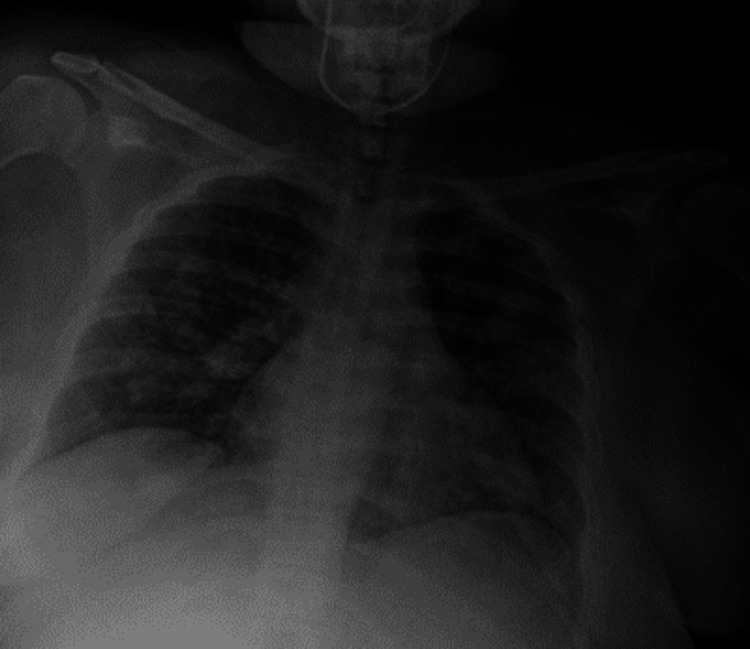
Chest X-ray image taken in the anteroposterior projection revealing scattered and diffuse fine reticular lung infiltrates in the periphery.

A 28-year-old female with a comprehensive vaccine history for influenza and COVID-19 and a long-standing asthma diagnosis using short-acting beta-2 agonist medication as rescue therapy 3-4 times a month, averaging one exacerbation per year, and a recent intranasal cocaine use (24 hours before), presented to the Emergency Department (ED). She reported a five-day progressing dyspnea, chest tightness, constant holo-cranial stabbing headache, and a persistent nonproductive cough. Initial vital signs were 36.3°C, blood pressure of 110/70, heart rate of 132 beats per minute (bpm), and oxygen saturation (SpO_2_) of 76%. The electrocardiogram revealed sinus tachycardia with no other abnormalities, and high-sensitivity troponins yielded normal values. Physical examination revealed accessory respiratory muscle use and wheezing. We started asthma exacerbation management with IV 40 mg methylprednisolone once per day and nebulized albuterol/ipratropium for an hour and then every hour. Initial laboratory tests were unremarkable. Influenza and SARS-CoV-2 tests returned negative results. Chest X-rays displayed patchy alveolar infiltrates, leading to the initiation of treatment for community-acquired pneumonia (CAP) with ceftriaxone and clarithromycin (Figure 1).

**Figure 2:**
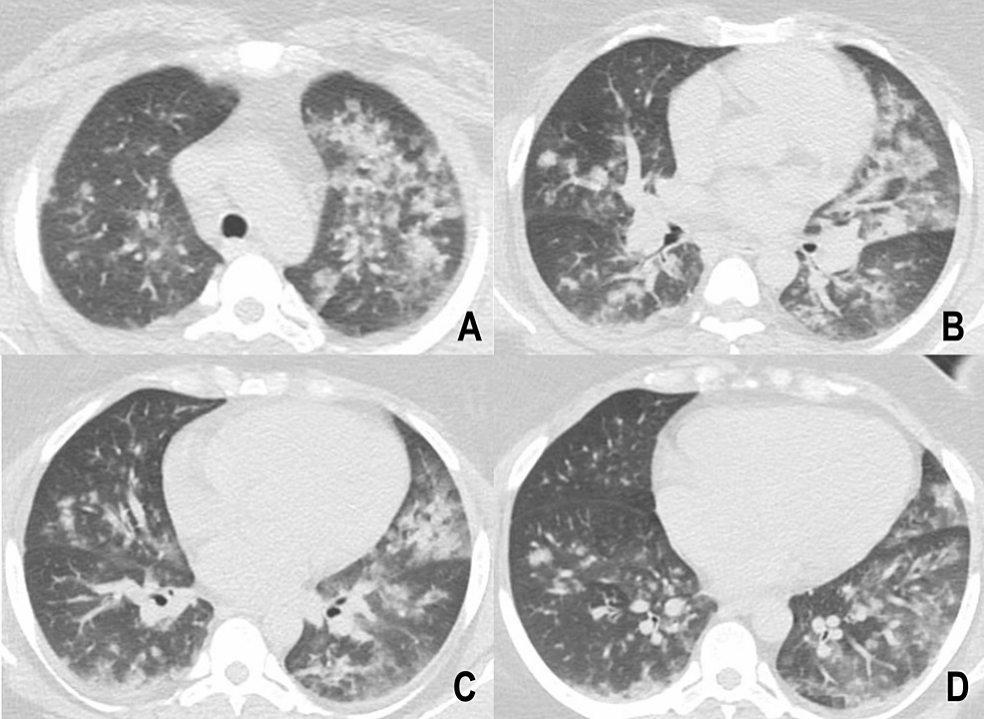
High-resolution tomography. (A) Ground glass opacities consolidate in the upper left lobe, in addition to separate areas with ground glass opacities. (B) Centrilobular nodules with nutrient vessels and areas with a mosaic pattern in the middle lobe. (C and D) Bilateral lower lobes present multiple ground glass centrilobular nodules, and the lower left lobe shows ground glass opacities and a mosaic pattern.

The patient continued to experience persistent coughing and thoracic pain despite initial management. This prompted a high-resolution CT evaluation to investigate other potential causes of lung damage, as seen in Figure 2.

**Figure 3:**
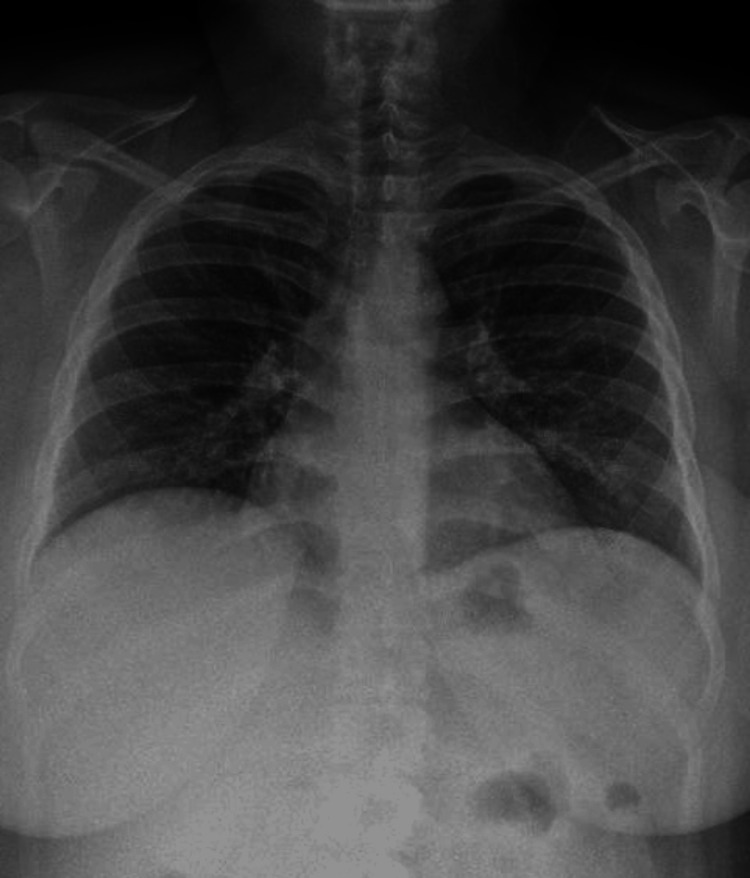
Chest X-ray, anteroposterior projection, showing the resolution of pulmonary infiltrates.

Bronchoscopy testing showed negative results for gram, acid-fast bacilli, multiple viral agent polymerase chain reaction (PCR), and potassium hydroxide tests. The cytology report showed 963 white blood cells, with 32% lymphocytes, 47% neutrophils, 4% eosinophils, and greater than 20% hemosiderin-laden macrophages. Oxygen needs resolved after 48 hours, and bacterial cultures yielded negative results. The patient fully recovered after four days of observation (Figure 3), allowing early hospital discharge. After analyzing this patient's clinical, radiological, and histopathological features, we determined that it resembled the CLS.

## Discussion

The strengths of this case report rest in its detailed exploration of clinical and radiological findings, providing valuable insights for emergency and hospital practitioners. The robust discussion highlights the nuanced relationship between asthmatic exacerbations and cocaine use. However, a notable limitation arises from the absence of histopathological image demonstration because of hospital-patient confidentiality. The lack of pulmonary function tests before and after the reported event hinders a more enriched discussion.

The term "crack lung" describes an acute pulmonary syndrome resulting from the inhalation of crystalline cocaine. Standard CLS features include fever, hypoxemia, hemoptysis, respiratory failure, and diffuse alveolar infiltrates densely populated with eosinophils. Radiological manifestations may include alveolar hemorrhage, hypersensitivity pneumonitis, and acute respiratory distress syndrome [5]. Kissner et al. introduced this term, noting increased immunoglobulin E levels in a patient with persistent cough and dyspnea, becoming relevant in drug-related allergic disease exacerbation [3].

Cocaine induces pulmonary damage through various mechanisms, including alveolocapillary membrane lesions, increased alveolar permeability, diffuse interstitial damage, and the generation of reactive oxygen species. The sympathomimetic effects on pulmonary vasculature lead to pulmonary vasoconstriction, and the repeated increase in pulmonary vascular tone translates into intermittent elevations in pulmonary pressure, which could lead to pulmonary embolism mimicry and anoxic lung injury. Microvasculature damage increases the release of endothelin-1 (ET-1), translating into vasoconstriction and the vascular leaking of fluid and erythrocytes to the alveolar space; this can be related to CLS presenting with alveolar hemorrhage and pulmonary edema. Cocaine induces severe inflammatory activity with an increase in polymorphonuclear cells and interleukin-8; in chronic users, this exposure can lead to fibrosis, metaplastic epithelium, alveolar wall damage, and emphysema. Inhaled products may cause mucosal damage and perforation in the larynx. Macrophage activity against tumors and bacteria decreases because of a diminished capability to generate reactive molecules [4,6].

Bender demonstrated a statistically significant increase in asthma symptoms in drug-using asthmatic patients (5.8% versus 3.7%) [7]. Tashkin et al. evaluated the airway dynamics of cocaine IV users against inhaled drug users, finding a significant decrease in airway conductance in the inhaled cocaine group, possibly attributed to airway irritation [8]. There may be exceptional cases where non-asthmatic patients present with an asthma-like CLS clinical presentation with severe obstructive patterns [9,10]. Cocaine has been associated with a threefold increase in the prevalence of new-onset bronchospasm or asthma exacerbation, extended hospital stays, intubations, and intensive care unit admissions due to asthma exacerbation. Our patient did not experience complications or require mechanical ventilation despite the potential for illicit drugs such as cocaine to trigger asthma crises [11,12].

Rubin and Neugarten published a report on six patients with long-standing asthma who experienced severe exacerbations due to cocaine use; all these patients responded well to standard treatment with bronchodilators and steroids. However, our patient had a slower response and showed multiple lung abnormalities on chest X-ray and CT scan [13].

Self et al. [14] conducted a review on the relationship between asthma and drug use, including cocaine. They mention 18 cases of asthmatic exacerbation with previous cocaine use, which included the ones reported by Rubin and Neugarten [13] and Kissner et al. [3], and three fatal asthma case series studies found that cocaine use was prevalent in 13% of 39 patients, 38% of 29 patients, and 44 cases of deaths [14]. Our case survived and had a prompt recovery with an early hospital discharge.

Radiological evaluation is nonspecific. No CT scan finding can confirm a CLS. Cocaine exposure can trigger eosinophilic lung disease, which causes ground glass opacities, nodules, and mosaic patterns. Our patient exhibited diffuse ground glass opacities, centrilobular nodules, and mosaic patterns [5].

The histopathological characteristics of "crack lung" include alveolar hemorrhage, congestion, edema, pneumonitis, interstitial fibrosis, small artery medial hypertrophy, hemosiderin-laden macrophages, and, in rare cases, carbon-pigmented macrophages and hemosiderin [15]. Our patient presented with an increase in all types of blood cells, including neutrophils, lymphocytes, eosinophils, and hemosiderin-laden macrophages.

There is no consensus on the best treatment for the acute asthma-like presentation of "crack lung." The previously mentioned cases have reported drug abstinence and standard asthma exacerbation therapy (steroids, beta-agonists, and oxygen therapy) as their management with no specific difference. Further investigations are necessary to understand this topic.

## Conclusions

The patient had a history of cocaine use and was experiencing asthma symptoms that were not responding to standard treatment. Upon conducting a chest CT scan, we found ground glass opacities and centrilobular nodules. Cytology from lung tissue suggested that the patient suffered from CLS. The presence of hemosiderin-laden macrophages further supported this diagnosis.

It is noteworthy that cocaine-induced lung damage can mimic or exacerbate several pulmonary pathologies, including asthma exacerbation, as exemplified in our case. It is paramount for clinicians to keep a high diagnostic suspicion for CLS when faced with patients with illicit drug use and persistent asthma crises. The lack of high-quality data for diagnosing and treating acute obstructive lung presentations of CLS emphasizes the need for further investigation in this area.
